# Long non-coding RNA PCDRlnc1 confers docetaxel resistance in prostate cancer by promoting autophagy

**DOI:** 10.7150/jca.65329

**Published:** 2022-04-04

**Authors:** Jianjun Xie, Xiumei Chen, Weiwan wang, Zhenghui Guan, Jianquan Hou, Jianzhong Lin

**Affiliations:** 1 Department of Urology, The First Affiliated Hospital of Soochow University, China.; 2 Department of Urology, The Affiliated Suzhou Hospital Hospital of Nanjing Medical, University, China.; 3 Department of Geriatrics, The First Affiliated Hospital of Nanjing Medical University, China.; 4 Central Laboratory, BenQ Medical Center, The Affiliated BenQ Hospital of Nanjing, Medical University, China.; 5 Department of Urology, Taizhou Clinical Medical School of Nanjing Medical University, China.

**Keywords:** LncRNA, Prostate cancer, Chemoresistance, Autophagy, UHRF1

## Abstract

Docetaxel resistance seriously affects its clinical application in prostate cancer (PCa). Long noncoding RNAs (lncRNAs) influence the chemosensitivity of various cancers. However, the potential involvement of lncRNAs in docetaxel sensitivity remains largely unknown in PCa. In the present study, we used RNA sequencing to compare the expression profiles of lncRNAs in docetaxel-resistant PCa cells and their parental cells and identified a novel lncRNA, ENSG00000234147, termed as PCa docetaxel resistance-associated lncRNA1 (PCDRlnc1). Our results indicated that PCDRlnc1 is closely associated with docetaxel resistance in PCa, and PCDRlnc1 knockout markedly sensitized the resistant cells to docetaxel *in vitro* and *in vivo*. In addition, PCDRlnc1 inhibition markedly suppressed docetaxel-induced autophagy. Conversely, PCDRlnc1 overexpression promoted autophagy. Mechanistically, PCDRlnc1 interacted with UHRF1 (ubiquitin-like with plant homeodomain and ring finger domains 1) and promoted its transcription level in PCa cells, leading to the activation of autophagic Beclin-1 signaling. Together, our data demonstrate that PCDRlnc1 is a novel key regulator of PCa docetaxel resistance, suggesting that it may be used as a potential biomarker of docetaxel resistance and therapeutic target in PCa.

## Introduction

Prostate cancer (PCa) is currently the most frequently diagnosed cancer in American males and the second leading cause of cancer-related deaths, with the incidence surging in China in recent years 1,2. Docetaxel, as the first-line standard chemotherapy method in advanced PCa, offers a limited survival advantage due to the development of drug resistance 3. Thus, elucidation of the underlying mechanisms of docetaxel resistance in such patients is required to develop new potentially effective treatments.

Autophagy is a cellular process that involves the disposal and recycling of intracellular misfolded proteins and malfunctioning organelles. It promotes tumor cell survival under therapeutic or metabolic stress 4. Recently, autophagy was confirmed to be an important mechanism of resistance to various chemotherapeutic agents, including docetaxel 5-8.

Pharmacological or genetic inhibition of autophagy has been demonstrated to improve the efficacy of chemotherapy for many cancers 9-11. Several studies have also verified that autophagy is involved in docetaxel resistance, and its inhibition can markedly improve the sensitivity of PCa cells to docetaxel 12-14. However, the role and potential mechanisms of autophagy-mediated docetaxel resistance in PCa remain to be elucidated.

Long non-coding RNAs (lncRNAs), a novel class of RNAs longer than 200 nucleotides without encoding for any protein, have been confirmed to play crucial roles in multiple cellular processes of cancer, including chemotherapy resistance 15-17. Some lncRNAs function as competitive platforms for cis- or trans-regulation, and their target genes are crucial regulators of autophagy-regulatory networks 18. LncRNAs are also implicated in the modulation of docetaxel resistance in PCa 19-21. Moreover, many lncRNAs can affect chemoresistance by regulating autophagy 9,10,22. However, the relation between lncRNA level and docetaxel insensitivity induced by autophagy is still elusive in PCa.

In this study, we screened the differentially expressed lncRNAs by RNA sequencing between parental PCa cells and docetaxel-resistant PCa cells. Among the upregulated lncRNAs, we identified a novel lncRNA ENST00000234147, which was associated with the chemoresistance of PCa cells *in vitro* and *in vivo*, and termed it as PCa docetaxel resistance-associated lncRNA1(PCDRlnc1). In addition, we found that PCDRlnc1 induces the chemoresistance of PCa by triggering autophagy. Mechanistically, lncRNA pulldown combined with mass spectrometry analysis showed that PCDRlnc1 could interact with UHRF1 protein and increase its level by promoting transcription, which further results in the improved level of Beclin-1 and eventually promotes protective autophagy to reduce the efficacy of docetaxel.

## Materials and methods

### Cell culture and reagents

Two CRPC cell lines, PC3 and DU145, were obtained from the Chinese Academy of Sciences, Shanghai Institute of Biochemistry and Cell Biology (Shanghai, China). Docetaxel-resistant cell lines (PC3-DR and DU145-DR) were developed according to our previous study 23. Briefly, resistant cell lines were cultured over 8 months by increasing the dose of docetaxel in a stepwise manner. Docetaxel and chloroquine (CQ) were purchased from Sangon Biotech (Shanghai, China) and MCE (Shanghai, China), respectively.

### Patient samples

Twenty-three metastatic (n=23) PCa tissue or blood samples were collected from patients undergoing palliative transurethral resection, radical prostatectomy or prostate biopsy in Suzhou Municipal Hospital and Nanjing BenQ Hospital between July 2017 and December 2020. All patients had received docetaxel-based chemotherapy. Prostate specific antigen (PSA) was evaluated once a month during treatment and follow-up.

Imaging was performed before treatment with docetaxel, when progression disease was suspected. Patients suffering from other tumor diseases or receiving other antitumor treatments were excluded. Among them, the number of docetaxel-sensitive patients and docetaxel-resistant patients was 13 and 10, respectively. The experiments were approved by the Ethics Committee of the Affiliated Nanjing BenQ Hospital of Nanjing Medical University.

### RNA transcriptome sequencing

RNA transcriptome sequencing was performed as previously described 10. The threshold set for downregulated and upregulated genes was a fold change >2.0 and a p-value <0.05. Clustering and combined analyses were conducted.

### Cell transfection

Transfections were performed using Lipofectamine 2000 reagent (Invitrogen, USA) according to the manufacturer's instructions. The vector pcDNA3.1-PCDRlnc1 was purchased from GenePharma. Beclin-1 and UHRF-1 siRNAs (GenePharma, China) were used to knock down their gene expression. The sense sequences of siRNAs were as follows: Beclin-1: 5'-CAGTTTGGCACAATCAATA-3'; URHF1: 5'-GCGCUGGCUCUCAACUGCU-3'. The sequence of non-targeting control siRNA (si-NC) was 5'-UUCUCCGAACGUGUCACGUTT-3'. The efficiency was assessed after 48h using qRT-PCR or WB. CRISPR-Cas9 for PCDRlnc1 knockout CRISPR/CAS gene-editing technology mediated by electroporation was used to knockout PCDRlnc1 in PC3-DR cells (Cyagen, China). The loss of PCDRlnc1 in PC3-DR cells was verified by PCR and sequencing Figure S1 and Figure S2.

### RNA extraction and quantitative real-time PCR (qRT-PCR)

Total RNA extraction and subsequently qRT-PCR were carried out as previously described 13. The primers are listed as follows: PCDRlnc1 (F-TGCCTGGCTAATGTTGGT, R-GTATTTGGAGGCTGGGTG), UHRF1 (F-ACCCCGACTCCTTAGAGCAT, R-CCCTGTAGAACAGCCTCTGC), Beclin-1 (F-AACCTCAGCCGAAGACTGAA, R-CAGTGACGTTGAGCTGAGTG), U6 (F-CTCGCTTCGGCAGCACA, R-AACGCTTCACGAATTTGCGT), and GAPDH (F-AGCCACATCGCTCAGACAC, R-GCCCAATACGACCAAATCC).

### Rapid amplification of cDNA ends (RACE)

Full-length amplification of PCDRlnc1 was carried out using the SMART RACE cDNA amplification kit (Cat. 634858, Clontech, Palo Alto, CA, USA) according to the manufacturer′s instructions. The primers used for RACE were as follows: 5'-RACE external (AGCACTTTGGGAGGCCAAAGCAG), 5'-RACE internal (CGAGCCAGCCTGACCAACATTAG), 3'-RACE external (GAGCCCAAGAAGCCAGAGTCTCATTGTG), and 3'-RACE internal (AAGCAGTTCTCCTAGATCAGCATCAG).

### Colony formation and cell viability assays

Details of colony formation and CCK8 assays are available in our previous report 13.

### Apoptosis analysis

Non-adherent and adherent cells were collected, and apoptosis was quantified using a FITC Annexin V apoptosis detection kit following the manufacturer's instructions.

### Western blot analysis (WB)

After the respective treatments, 1×10^6^ cells were lysed using RIPA buffer mixed with protease inhibitors (Sigma, St Louis, MO, USA) and phosphatase inhibitors (Roche, Penzberg, Germany). A total of 20 μg of protein from each sample was mixed with 5×Lane Marker Reducing Sample Buffer (Pierce, Rockford, USA) and separated on a 10% SDS-polyacrylamide gel. Then, the protein bands were transferred to PVDF membranes and incubated with different dilutions of primary antibodies overnight at 4 °C. Following three washes, the membranes were incubated with secondary antibody overnight at 4 °C. Signals were visualized by using an ECL kit. Specific antibodies against p62 (1:2000, Abcam), LC3 (1:2000, Abcam), ATG5 (1:1000, Abcam), ATG7 (1:5000, Abcam), AMPK or p-AMPK (1:2000, Abcam), MTOR or p-MTOR (1:2000, Abcam), Beclin-1 (1:2000, Abcam), and GAPDH were used. Finally, the proteins were examined by using chemiluminescent reagents and Chemstudio plus imaging system.

### Confocal microscopy

Cells were first prepared on slides, transfected with GFP-RFP-LC3 adenovirus, and treated as indicated. After treatment with PBS buffer, the cells were then fixed with 4% paraformaldehyde. The slides were subsequently blocked with glycerol, and the localization of LC3 puncta was visualized using a confocal fluorescence microscope.

### Transmission electron microscopy (TEM)

TEM was performed as previously described 13. Images were acquired using a CM-120 electron microscope (Tecnai Sprite Biotwin, FEI).

### RNA pull-down and mass spectrometry (MS) analysis

Briefly, PCDRlnc1 RNAs were labeled with desthiobiotin using the Pierce RNA 3' end desthiobiotinylation kit, and RNA pull-down assays were performed according to the manufacturer's instructions (Cat.20164, RNA -protein pull-down kit, Components, Thermo). After elution of lncRNA-interacting proteins, the samples were subsequently subjected to mass spectrometric analysis.

### RNA immunoprecipitation (RIP)

In brief, RIP experiments were performed using a Magna RIP™ RNA-binding protein immunoprecipitation kit (Millipore, USA) following the manufacturer's instructions. The antibody against UHRF1 for the RIP assay was purchased from Abcam.

### Xenograft studies

Briefly, PC3-DR or PCDRlnc1-deleted PC3-DR cells were injected into the flanks of Balb/c nude mice (male, 6-8 weeks). After the tumors reached about 100 mm^3^, docetaxel (10 mg/kg) was administered intraperitoneally twice a week. The tumor volume was monitored regularly, and the mice were killed after 1 month. The tumors were removed, and a portion of the tumor tissues was immediately frozen in liquid nitrogen for protein detection or fixed in formalin for TUNEL assay. The animal experimental protocol was approved by the Ethics Committee of Nanjing BenQ Hospital, and experiments conformed to all relevant regulatory standards.

### TUNEL assay

To evaluate apoptosis in tumor tissues, a TUNEL assay was conducted as previously described 23. The tumor tissue sections were analyzed by fluorescence microscopy (Olympus, Japan).

### Statistical analysis

Data are presented as the mean±standard deviation. Statistical analysis was performed using GraphPad Prism 8.2.1. Differences between groups were analyzed by the Student t-test or two-way ANOVA. All statistical tests were two-sided, and P<0.05 was considered to indicate a statistically significant difference.

## Results

### Identification of PCDRlnc1 in docetaxel-resistant PCa

**Figure 1:**
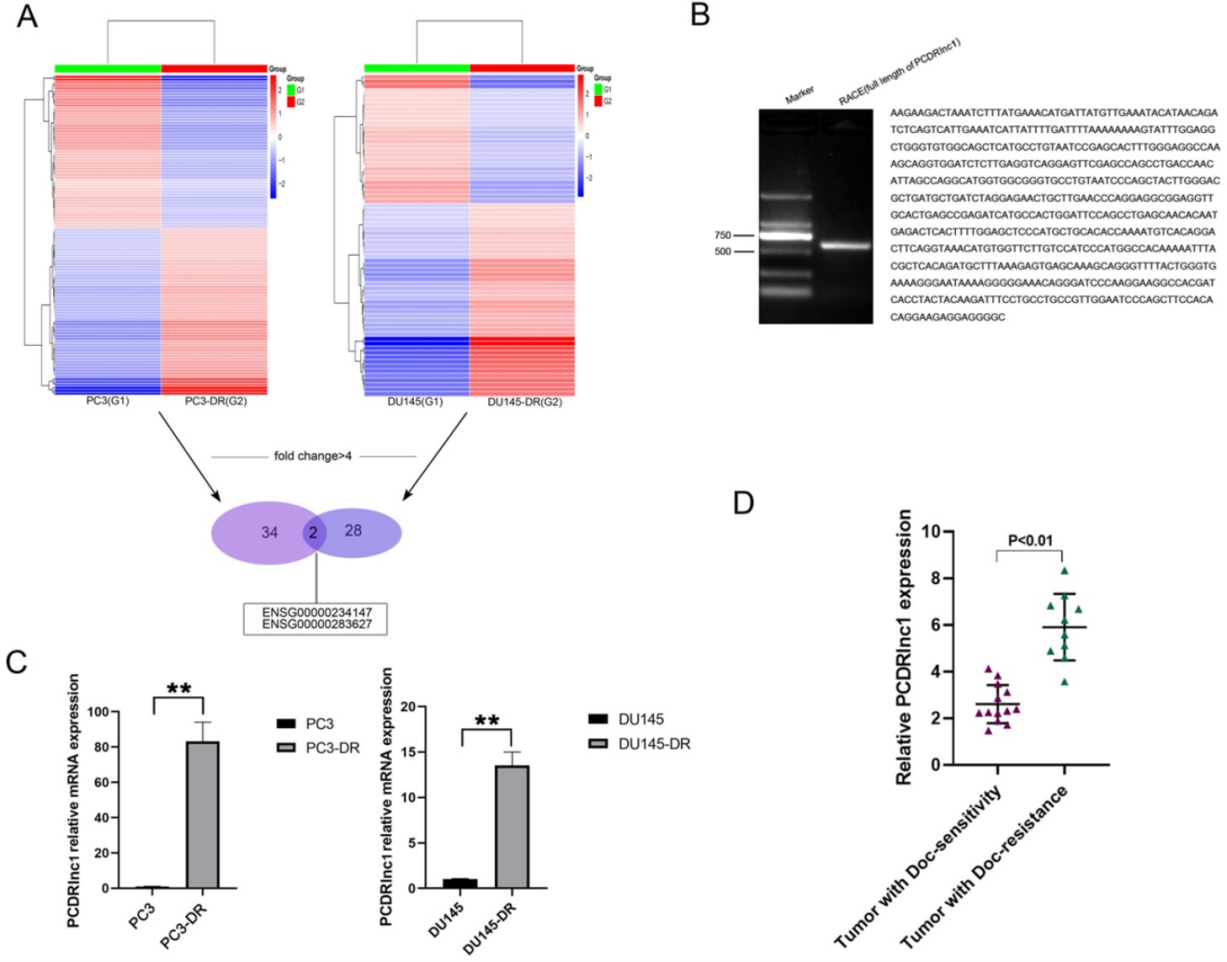
** Identification of PCDRlnc1 in docetaxel-resistant PCa. (A)** The cluster heat map indicates the differentially expressed lncRNAs. The red and green colors show high and low expression levels, respectively. Two lncRNAs (ENSG00000234147 and ENSG00000283627) were screened (fold change>4) in both PC3-DR and DU145-DR cells. **(B)** The full sequence of PCDRlnc1 was confirmed by RACE. **(C)** Relative expression of ENSG00000234147 (prostate cancer docetaxel resistance-associated lncRNA1, PCDRlnc1) in resistant cells and their parental cells was detected by qRT-PCR. **(D)** PCDRlnc1 expression was detected in docetaxel-resistant resistant PCa samples (n=10) and compared with that in docetaxel-sensitive PCa samples (n=13) using qRT-PCR analysis. ^*^P<0.05 and ^**^P<0.01, compared with the control group.

To identify lncRNAs attributed to docetaxel resistance in PCa, RNA sequencing was utilized to compare lncRNA expression profiles between parental (PC3 and DU145) and docetaxel-resistant PCa cells (PC3-DR and DU145-DR). Heatmaps suggested a clear distinction between parental and resistant cells. The number of most deferentially expressed lncRNAs (fold change >4) in PC3-DR and DU145-DR cells was 34 and 28, respectively, and two lncRNAs (ENSG00000234147 and ENSG00000283627) were found among them (Figure 1A). RACE allows us to quickly obtain full-length cDNA when the sequence is only partially known. In the present study, RACE-PCR and gene sequencing were performed in PC3-DR cells, since no related studies of ENSG0 0000234147(PCDRlnc1) have been reported. The full-length sequence was retrieved (594bp, Figure 1B). The results of qRT-PCR further confirmed the difference in the expression of PCDRlnc1 from high-throughput data (Figure 1C). Markedly increased expression of PCDRlnc1 was also observed in docetaxel-resistant PCa samples as compared to docetaxel-sensitive PCa samples by qRT-PCR analysis (Figure 1D).

### PCDRlnc1 promotes docetaxel resistance in PCa

**Figure 2:**
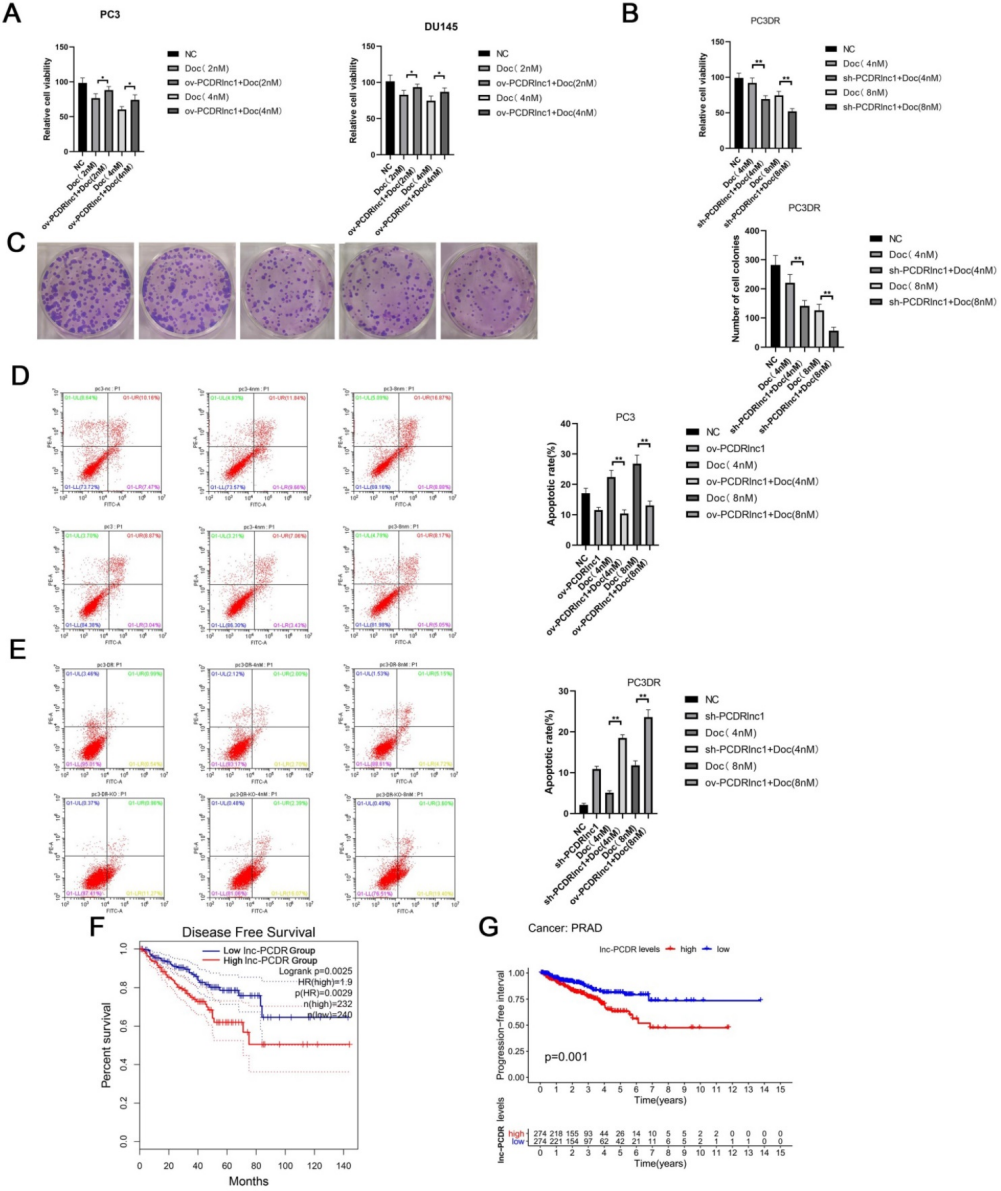
** PCDRlnc1 promotes docetaxel resistance in PCa. (A)** CCK8 assays were performed to determine cell proliferation after 2 nM and 4nM docetaxel (Doc) treatment for 48h in the plasmid-mediated PCDRlnc1 overexpression (ov-PCDRlnc1) PC3 and DU145 cells. Cas9-mediated PCDRlnc1 knockout (sh-PCDR lnc1) PC3-DR cells were treated with Doc (4 nM and 8 nM) for 48 h, and cell viability was measured using the CCK8 assay **(B)** and colony formation assay **(C).** Flow cytometric analyses were conducted to assess the effect on the apoptosis of ov-PCDRlnc1 **(D)** and sh-PCDRlnc1 **(E)** under the treatment of docetaxel (4 nM and 8 nM) for 24h in PC3 and PC3-DR cells. Representative images and the quantification are presented**,** respectively. Kaplan-Meier analyses of the relationship between PCDRlnc1 expression and disease-free survival (DFS) **(F)** or progression-free survival (PFS) **(G)** in PCa patients in the TCGA cohort. The data are presented as the mean±SD of three independent experiments. ^*^P<0.05 and ^**^P<0.01, compared with the control group.

To further confirm the function of PCDRlnc1, Cas9-mediated knockout (sh-PCDRlnc 1) vectors and plasmid-mediated overexpression (ov-PCDRlnc1) were used for exogenously manipulating the level of PCDRlnc1 in PCa cells (Figure S1-2 and Figure S3). The CCK8 assay showed that PCDRlnc1 overexpression suppressed the cytotoxicity of docetaxel in PC3 and DU145 cells (Figure 2A), whereas PC3-DR cells with PCDRlnc1 knockout showed greater sensitivity than control cells to different concentrations of docetaxel (Figure 2B). Consistently, the monolayer colony formation assay confirmed that PC3-DR shPCDRlnc1 cells formed fewer colonies than control cells under docetaxel treatment (Figure 2C). Furthermore, flow cytometric analysis was used to assess whether PCDRlnc1 influenced the apoptosis of PC3-DR cells. The results indicated that PCDRlnc1 knockout promotes docetaxel-induced cell apoptosis (Figure 2D), whereas PCDRlnc1 overexpression in wild-type PC3 cells showed the opposite effect after exposure to different concentrations of docetaxel (Figure 2E). Moreover, Kaplan-Meier analysis indicated that patients with high PCDRlnc1 levels have a markedly shorter disease-free survival period (Figure 2F) and progression-free interval (Figure 2G) than those with low PCDRlnc1 expression. Taken together, the above data indicated that PCDRlnc1 promotes docetaxel resistance, and its silencing makes PCa cells more sensitive to docetaxel.

### PCDRlnc1 promotes autophagy in PCa

**Figure 3:**
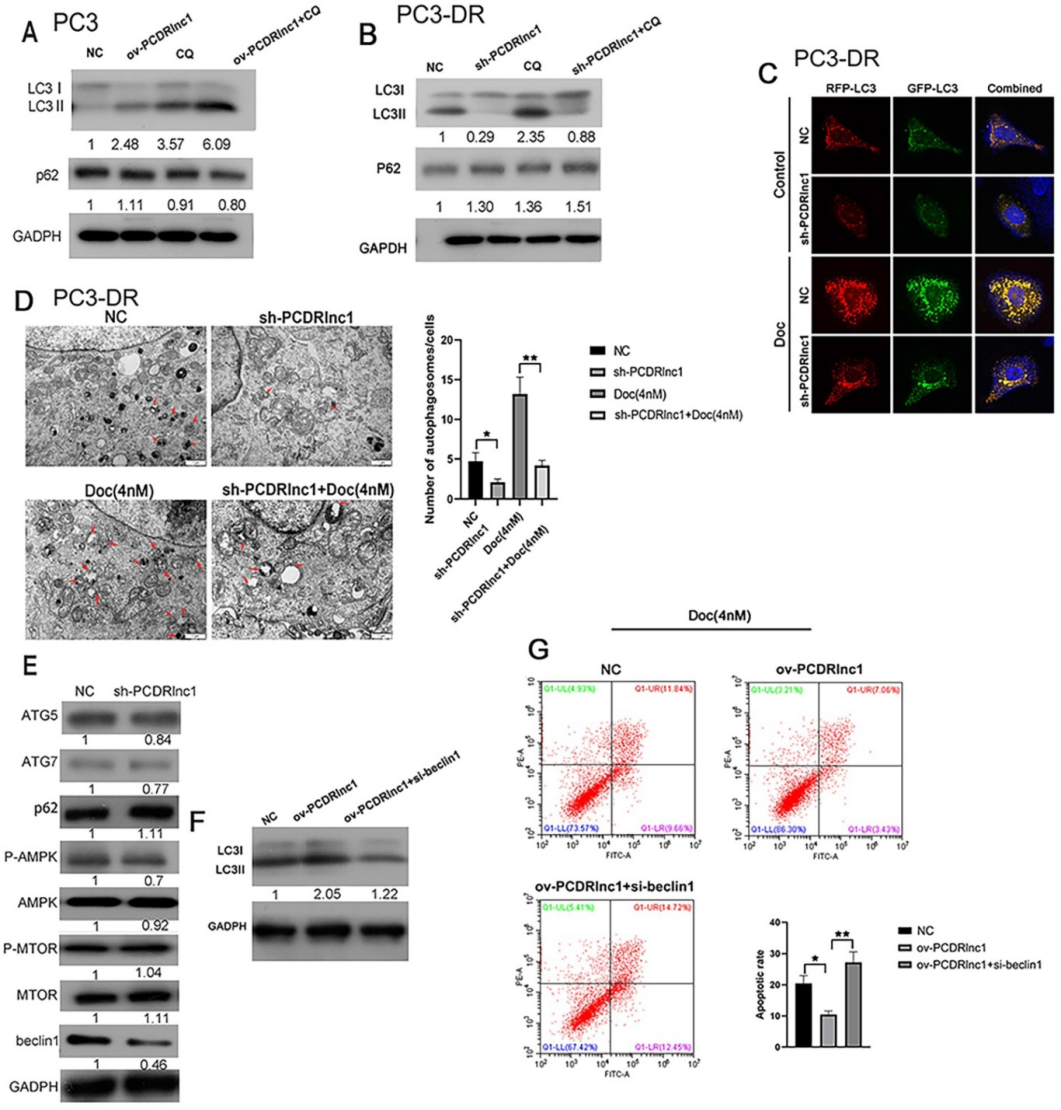
** PCDRlnc1 promotes autophagy to decrease docetaxel sensitivity in PCa.** Western blotting was performed to detect the protein levels of LC3B and p62 in PCDRlnc1 overexpression (ov-PCDRlnc1) PC3 **(A)** or PCDRlnc1 knockout (sh-PCDRlnc1) PC3-DR cells **(B)** under treatment with chloroquine (CQ;10 µM). **(C)** Confocal microscopy showing the effects of PCDRlnc1 knockout alone or in combination with 4 nM Doc in PC3-DR cells on mRFP-GFP-LC3 distribution after mRFP-GFP-LC3 adenovirus transfection. A representative image from three independent experiments in every group is shown. **(D)** The ultrastructure of shPCDRlnc1 PC3-DR cells was observed by transmission electron microscopy (TEM) after treatment with 4 nM docetaxel for 24 h. Representative images are presented. The number of autophagosomes observed by TEM was calculated. Scale bar, 1 µm. **(E)** The protein levels of p62, ATG5, ATG7, AMPK, p-AMPK, mTOR, p-mTOR, and Beclin-1 in PC3DR and sh-PCDRlnc1 PC3DR cells were determined by western blotting. PC3 cells were subjected to the simultaneous overexpression of PCDRlnc1 and interference of Beclin-1. Western Blotting was used to detect the expression of LC3B **(F),** and flow cytometric analysis was adopted to examine apoptosis under the treatment of Doc (4 nM) **(G).** ^*^P<0.05 and ^**^P<0.01, compared with the control group.

Recent studies have reported that lncRNAs can regulate autophagic activity to influence the chemotherapeutic efficacy of drugs 9,10,22. In this study, we also explored whether PCDRlnc1 influenced autophagy in PC3 cells and its resistant variant (PC3-DR). The levels of LC3B and p62, which are widely applied markers of autophagic flux, were detected by WB. The protein expression of LC3B-II was found to be markedly increased in PCDRlnc1-overexpressing PC3 cells, whereas decreased autophagic flux was observed in PCDRlnc1-deleted PC3-DR cells under CQ treatment, an autophagic lysosomal inhibitor (Figure 3A and 3B). Activation of autophagy often leads to the clearance of p62, however, in this study, neither PCDRlnc1 overexpression nor knockout markedly affected p62 protein levels (Figure 3A and 3B). Dual-fluorescence mRFP-GFP-LC3 can distinguish between autophagosomes and autolysosomes, wherein the GFP signal is vulnerable to acidic condition after autolysosome formation, and the mRFP signal is less affected. Therefore, the yellow puncta and red puncta indicate autophagosomes and autolysosomes, respectively. To further assess the role of PCDRlnc1 in regulating autophagic flux in cells, we exogenously introduced LC3B using the lentivirus system and performed tandem mRFP-GFP-LC3 fluorescence analysis, and then evaluated the results by observing under a laser scanning confocal microscope. The results verified that the numbers of both stubRFP-sensGFP-LC3 puncta and stubRFP-LC3 puncta were markedly reduced in shPCDRlnc1 PC3-DR cells, and the autophagic flux was also attenuated after docetaxel treatment, which normally induces autophagy (Figure 3C).

Consistently, TEM analysis of cellular ultrastructure indicated that autophagosome formation was suppressed in PCDRlnc1-deleted cells, especially after treatment with docetaxel (Figure 3D). To further examine how PCDRlnc1 regulates autophagy, we evaluated the effect of PCDRlnc1 knockout on several autophagy-related proteins (p62, ATG5, ATG7, mTOR, AMPK, and Beclin-1). The results showed the greatest change in Beclin-1 expression (Figure 3E). Based on these results, we next assessed the effect of the siRNA-mediated knockdown of Beclin-1 on PCDRlnc1-induced autophagy by assessing the level of LC3B protein. As expected, Beclin-1 knockdown blocked PCDRlnc1-induced increase in the LC3 level (Figure 3F). Moreover, we found that the suppression of PCDRlnc1 overexpression-induced apoptosis could be partly abolished by Beclin-1 inhibition (Figure 3G). Collectively, the above results suggested that PCDRlnc1 induces autophagy via Beclin-1 in PCa.

### PCDRlnc1 interacts with UHRF1 in PCa cells

**Figure 4:**
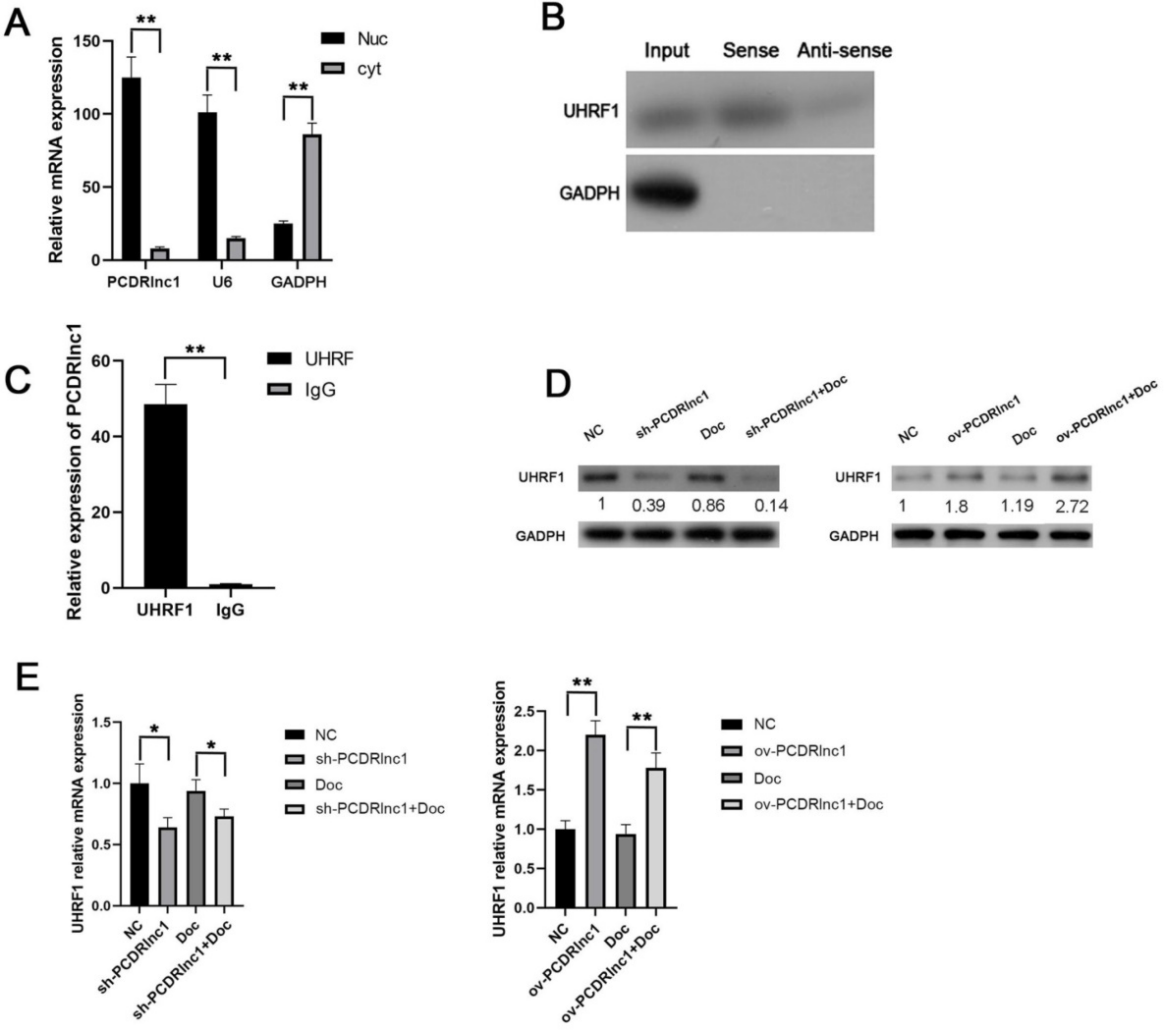
** PCDRlnc1 interacts with UHRF1 in PCa cells *in vitro*. (A)** After nuclear and cytosolic separation, RNA expression levels were measured by qRT-PCR. GAPDH and U6 were used as a cytosol marker and a nucleus marker, respectively. **(B)** RNA pulldown assay was performed using the biotin-labeled PCDRlnc1 probe, and the protein expression of UHRF1 expression was determined by western blotting. The antisense of the PCDRlnc1 probe was used as negative control. **(C)** RIP assay using anti-UHRF1 was performed in PC3-DR cells, and the enrichment of PCDRlnc1 mRNA was detected by qRT-PCR **(D** and** E).** Western blotting and qRT-PCR were performed to detect the protein and mRNA expression of UHRF1 when PCDRlnc1 was knocked out in PC3-DR cells or overexpressed in PC3 cells under the treatment of Doc (4 nM). The results are presented as the mean±SD of values obtained in three independent experiments. ^*^P<0.05 and ^**^P<0.01, compared with the control group.

To further elucidate the potential molecular mechanism by which PCDRlnc1 induces autophagy in PCa, we performed subcellular fractionation assay (Figure 4A) to evaluate the expression of PCDRlnc1 in the nucleus and cytoplasm in PC3-DR cells and found that PCDRlnc1 is mainly expressed in the nucleus, indicating that it may play a regulatory function at the transcriptional level. LncRNA pulldown combined with mass spectrometry has been often used to determine its interacting protein partners. Then, the RNA-related proteins were pulled down using biotin-labeled antisense- or sense-PCDRlnc1, and the proteins were identified by MS. Among the highly enriched proteins, we focused on UHRF1 due to its established role in chemotherapy resistance. WB was used to confirm the direct interaction between PCDRlnc1 and UHRF1 (Figure 4B). RIP further showed that UHRF1 could capture a significantly greater amount of PCDRlnc1 than the IgG antibody in PC3-DR cells (Figure 4C). Furthermore, qRT-PCR and WB analyses indicated a consistent trend in the mRNA and protein levels of UHRF1 when PCDRlnc1 was overexpressed in PC3 cells or was silenced in PC3-DR cells in the presence or absence of docetaxel (Figure 4D and E). Based on these results, we conclude that PCDRlnc1 may partially participate in making PCa cells resistant to docetaxel through the transcriptional regulation of UHRF1.

### PCDRlnc1 regulates autophagy-associated chemoresistance of PCa cells via UHRF1

**Figure 5:**
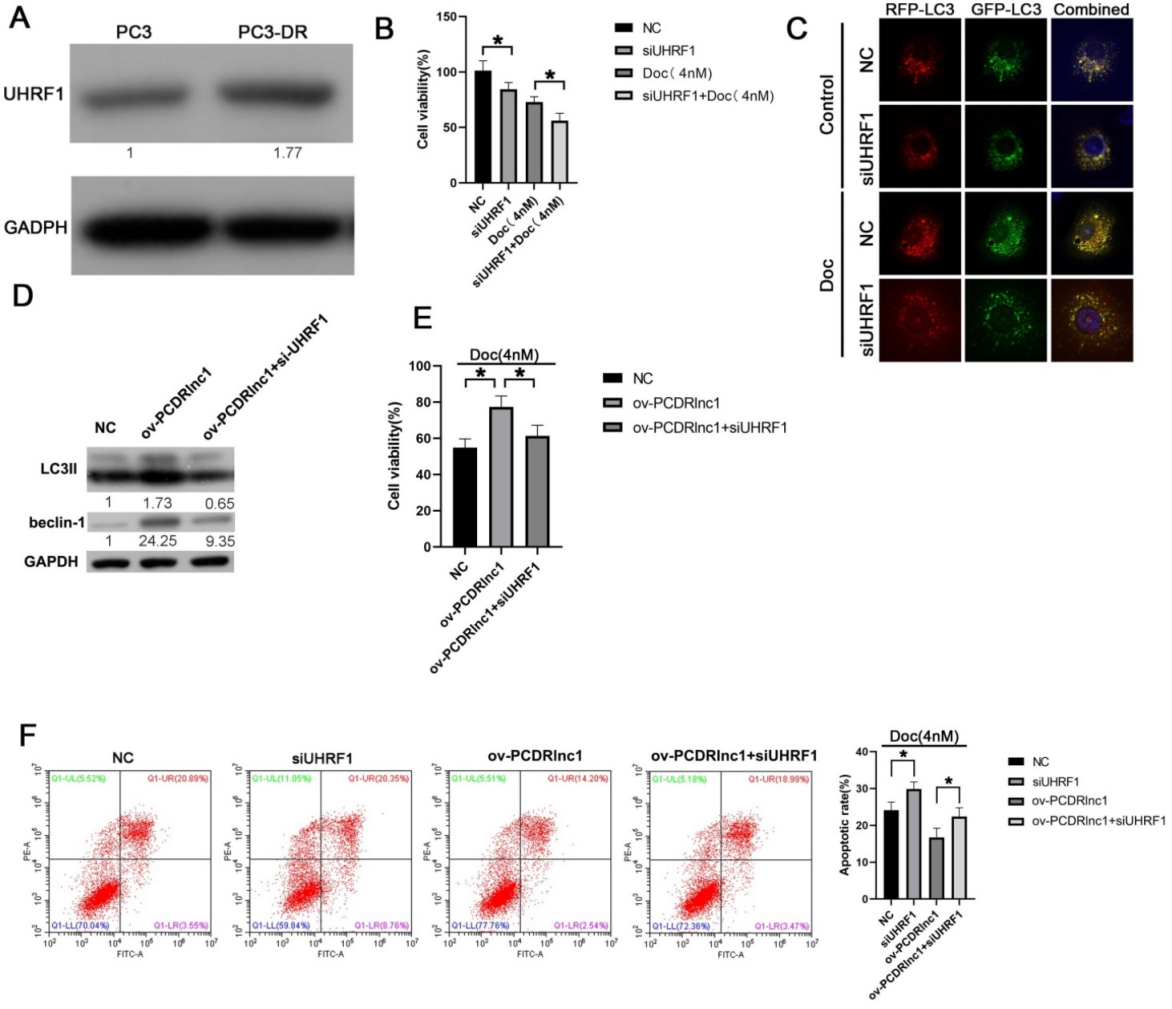
** PCDRlnc1 regulates autophagy-associated chemoresistance of PCa cells *in vitro* via UHRF1. (A)** The protein expression of UHRF1 was analyzed in PC3 and PC3-DR cells. **(B)** UHRF1 was inhibited by siRNA in PC3-DR cells, and the CCK8 assay was conducted to evaluate the effect of UHRF1 knockdown on docetaxel cytotoxicity. **(C)** PC3-DR cells stably expressing stubRFP-sensGFP-LC3 under UHRF1 knockdown were examined under a fluorescence microscope in the absence or presence of docetaxel (Doc, 4 nM). **(D)** PC3 cells were subjected to the simultaneous overexpression of PCDRlnc1 and interference of UHRF1 under the treatment of Doc (4 nM). Western blotting was used to examine the protein levels of LC3B and Beclin-1. CCK8 assays **(E)** and flow cytometric analyses **(F)** were used to detect cell proliferation and apoptosis, respectively. *P<0.05 and **P<0.01, compared with the control group.

In the present study, higher UHRF1 expression was found in PC3-DR cells than in PC3 cells (Figure 5A), and its siRNA-mediated inhibition markedly improved docetaxel sensitivity (Figure 5B and Figure S4). Furthermore, stubRFP-sensGFP-LC3 staining showed that UHRF1 knockdown could suppress the autophagic flux of PC3-DR cells (Figure 5C). Moreover, the increased expression of LC3II and Beclin-1 induced by PCDRlnc1 overexpression was reversed by treatment with UHRF1 siRNA at the protein level (Figure 5D). Additionally, knockdown of UHRF1 could reverse PCDRlnc1-mediated growth promotion and apoptosis inhibition under docetaxel treatment (Figure 5E and 5F). Taken together, these results indicated that PCDRlnc1 regulates Beclin1-mediated autophagy via UHRF1, which further makes PCa cells resistant to docetaxel. Knockout of PCDRlnc1 inhibits autophagy and improves the sensitivity of PCa cells to docetaxel *in vivo*.

**Figure 6:**
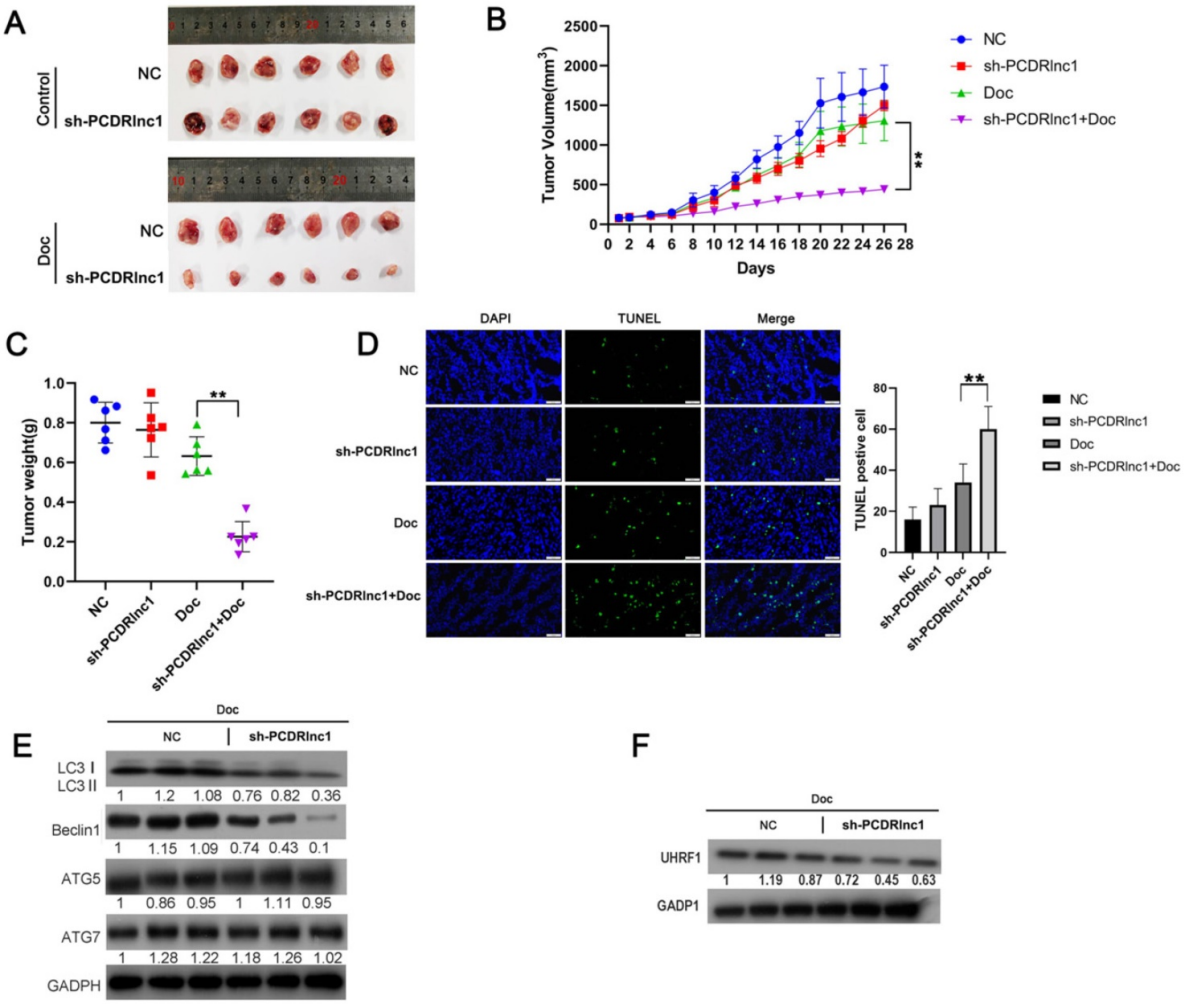
** PCDRlnc1 knockout inhibits autophagy and improves the sensitivity of PCa cells to docetaxel *in vivo*.** Tumor growth **(A and B)** and tumor weight **(C)** in the sh-PCDRlnc1 PC3DR and negative control (NC) groups with or without docetaxel (Doc) treatment. **(D)** Representative sections and the average number of TUNEL-positive cells in the subcutaneous tumors. Scale bar, 50 µm. **(E)** Western blotting was used to examine the protein expression of LC3II, ATG5, ATG7, and Beclin-1 in tumor tissues. **(F)** Western blotting was performed to assess the protein level of UHRF1 under the treatment of Doc. The results are presented as the mean±SD of values obtained in three independent experiments. *P<0.05 and **P<0.01, compared with the control group.

In our experiment to explore whether PCDRlnc1 affected the sensitivity of xenograft tumors to docetaxel *in vivo*, we found no significant difference in the growth of PC3-DR cell xenografts before and after PCDRlnc1 knockout without docetaxel treatment. However, PCDRlnc1-deleted xenografts grew more slowly than control xenografts when treated with docetaxel (Figure 6A and 6B). Moreover, docetaxel significantly inhibited tumor growth in the PCDRlnc1-deleted group compared with the control group (Figure 6C). More importantly, cell apoptosis after treatment with docetaxel was much higher in the PCDRlnc1-deleted group than in the control group (Figure 6D). To further evaluate the potential effect on autophagy *in vivo*, we performed WB to quantify the level of LC3B-II, ATG5, ATG7, and Beclin-1 and found that only LC3B-II and Beclin-1 levels were markedly suppressed in the PCDRlnc1-deleted group (Figure 6E). Additionally, the inhibition of PCDRlnc1 considerably decreased UHRF1 protein expression (Figure 6F).

## Discussion

Docetaxel-based chemotherapy has been extensively used as the standardized first-line therapeutic strategy for advanced PCa patients, but resistance to docetaxel limits its survival benefits. Therefore, it is essential to investigate the mechanisms underlying the acquired resistance to docetaxel. LncRNAs show distinct cellular localizations and a wide range of expression levels; thus, they regulate several cellular functions 24. Its regulatory role in the chemoresistance of various cancers, including PCa, has been well documented 10,19-22. However, the potentially relevant molecular mechanisms of chemoresistance in PCa remain elusive.

In recent years, some newly discovered lncRNAs have been reported to regulate the chemosensitivity of cancer cells. For example, lncGBCDR makes gallbladder cancer cells resistant to doxorubicin 9. LncARSR promotes sunitinib resistance in renal cancer 25. In the present study, we screened out a novel lncRNA (PCDRlnc1) by comparing docetaxel-resistant PCa cells with the parental cells, and the expression of PCDRlnc1 was subsequently confirmed to be markedly enhanced in docetaxel-resistant PCa cells and clinical samples. Furthermore, PCDRlnc1 overexpression promoted the proliferation and inhibited the apoptosis of PCa cells under docetaxel treatment. More importantly, silencing of PCDRlnc1 sensitized resistant cells to the treatment of docetaxel both *in vitro* and *in vivo*. Additionally, high PCDRlnc1 expression in PCa tissues was associated with a poor prognosis. These data demonstrate that PCDRlnc1 expression dictates the clinical efficacy of docetaxel treatment in PCa. However, the role of PCDRlnc1 has not been studied in a large cohort of samples, and future clinical studies are therefore needed to validate the role of PCDRlnc1.

The induction of autophagy can make PCa cells resistant to docetaxel, suggesting that its inhibition may benefit PCa therapy and prevention 12-14. Autophagy has been closely linked to the malignancy of PCa 26-29. Previous studies have confirmed that DU145 cells are refractory to autophagy activation because they lack full-length ATG5 30,31. Therefore, we chose PC3 cells to preliminarily evaluate the effect of PCDRlnc1 on their autophagy and the cytotoxicity of docetaxel in the present study. Through overexpression and deletion of function assays, we found that PCDRlnc1 modulates the chemosensitivity of PCa cells by altering the extent of autophagy *in vitro* and *in vivo*. Our previous study confirmed that the AMPK/mTOR pathway was associated with docetaxel resistance in PCa, but in this study, PCDRlnc1 failed to affect this pathway. The level of p62, a well-known autophagic substrate, remained unchanged upon overexpression or knockout of PCDRlnc1. P62 is reported to be transcriptionally and post-translationally regulated by numerous factors, but its expression is not related to autophagy 32,33. Remarkably, the expression of Beclin-1, an important autophagic component, was significantly decreased by silencing PCDRlnc1 and increased by overexpressing PCDRlnc1. In addition, the upregulation of autophagy by PCDRlnc1 was partially reversed by the suppression of Beclin-1, and the inhibition of docetaxel cytotoxicity induced by PCDRlnc1 overexpression could be recovered by Beclin-1 genetic inhibition. Therefore, we propose that PCDRlnc1 induces the docetaxel resistance of PCa cells by promoting Beclin-1-mediated autophagy. Of course, this effect of PCDRlnc1 needs to be verified in more cell lines and clinical samples.

LncRNAs can function as cis-acting RNAs to regulate the expression of neighboring genes or as trans-acting RNAs to play multiple roles 18. No adjacent genes were found to be regulated by PCDRlnc1, indicating that it may play a regulatory function in the trans mode. Currently, lncRNAs are considered to function by interacting with binding proteins 34. Therefore, we performed RNA pull-down, mass spectrometry, and RIP analyses and then identified UHRF1 to interact with PCDRlnc1. UHRF1 has been demonstrated to induce chemoresistance and radioresistance in several cancers, including PCa 35-37. Similarly, we found UHRF1 protein levels to be significantly higher in resistant PCa cells. We also showed that UHRF1 knockdown significantly suppresses PCDRlnc1-mediated autophagic flux and improves the docetaxel chemosensitivity of resistant cells. Thus, we propose that PCDRlnc1 promotes autophagy and docetaxel resistance in PCa via UHRF1.

UHRF1 can be regulated by the ubiquitin-proteasome pathway outside the nucleus 38-40 or at the transcriptional level in the nucleus 35,41. We next investigated how PCDRlnc1 promotes autophagy in PCa. In our study, PCDRlnc1 was found to be mainly located in the nucleus, suggesting that it functions at the transcriptional level. In line with this, our results revealed that the mRNA and protein levels of UHRF1 positively correlate with PCDRlnc1 expression. However, we should mention that although these results initially indicate that PCDRlnc1 may function to regulate UHRF1 gene transcription, the functional fragment and exact mechanism of PCDRlnc1 on UHRF1 transcriptional regulation needs to be further studied. Previous studies suggested that the increased expression of Beclin-1, a key mediator of autophagy, results in the development of docetaxel resistance in PCa, and its inhibition leads to the resensitization of docetaxel-resistant PCa cells 12,13. Our present data also demonstrated that PCDRlnc-induced Beclin-1 upregulation could be neutralized by UHRF1 knockdown, indicating that Beclin-1 is a downstream target of PCDRlnc1/UHRF1 pathway. However, it is reported that UHRF1 regulates the expression of associated targeted genes by regulating transcriptional 42,43 or posttranscriptional processes 44. Therefore, further in-depth studies would be needed to examine the mechanisms underlying the regulation of Beclin-1 in the PCDRlnc1/UHRF1 pathway, thus affecting the autophagy of PCa cells.

## Conclusion

**Figure 7:**
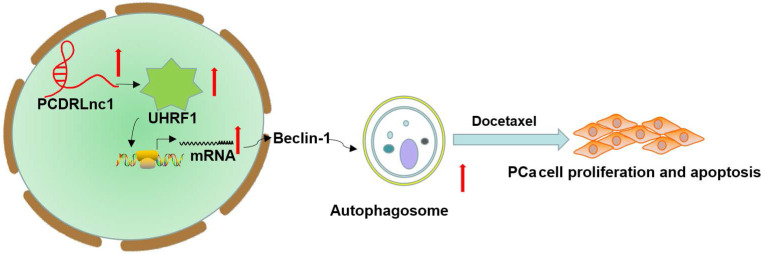
** Schematic representation of the effect of PCDRlnc1-mediated Beclin-1-dependent autophagy on docetaxel resistance in PCa.** PCDRlnc1 could interact with UHRF1. The transcriptional regulation of PCDRlnc1 on UHRF1 might positively regulate the expression of Beclin-1 and the subsequent autophagic process, which makes PCa cells resistant to docetaxel.

In summary, we identified that PCDRlnc1 promotes autophagy and docetaxel resistance of PCa cells by interacting with UHRF1 and promoting its transcription, which eventually upregulates Beclin-1 expression (Figure 7). Therefore, PCDRlnc1 may be further studied as a potential biomarker and an attractive target for the treatment of docetaxel-resistant PCa.

## Supplementary Material

Supplementary figures.

## Abbreviations

PCa: prostate cancer
Doc: docetaxel
lncRNA: Long noncoding RNA
UHRF1: ubiquitin-like with plant homeodomain and ring finger domains 1
PCDRlnc1: prostate cancer docetaxel resistance-associated lncRNA1
NC: negative control
RIP: RNA immunoprecipitation
RACE: Rapid amplification of cDNA ends
TEM: Transmission electron microscopy
MS: mass spectrometry
WB: Western blot
qRT-PCR: quantitative real-time PCR
