# Supplementary material for: Long non-coding RNA PCDRlnc1 confers docetaxel resistance in prostate cancer by promoting autophagy
Source: J Cancer. 2022 Apr 4;13(7):2138–49. doi: 10.7150/jca.65329 (PMC9066218; doi:10.7150/jca.65329)
Supplement: Supplementary file 1 — Supplementary figures. [file jcav13p2138s1.pdf]

Supplementary figure legends

Figure S1. PCR was used to identify if PCDRlnc1 knockout was homozygous

Figure S2. Verification of the deletion of PCDRlnc1 using sequencing

Figure S3. PCR was used to verify PCDRlnc1 overexpression

Figure S4. Western blotting was used to confirm the knockdown of UHRF1 at the protein level

PCR identified as homozygote

Multiple sets of primers were designed before and after the two target sites for PCR amplification. For the homozygotes with gene fragment knockout, Region 1 and Region 2 could not amplify corresponding fragments, only **Region 3** could amplify truncated fragments.

PCR identification site

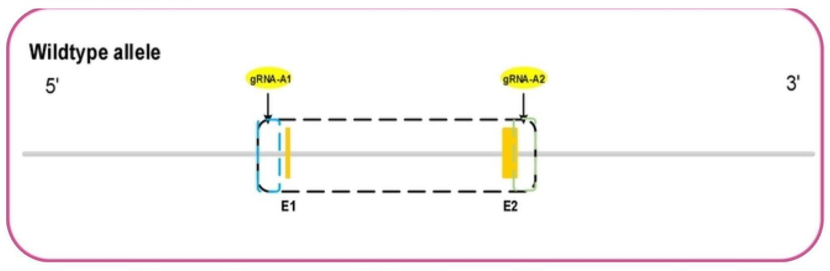

gRNA sequence for Cas 9 is as follows:

gRNA-A1:AGTATTCACCTACTGTAGCA

gRNA-A2:GGAGCTCCAGGCAACCTAGT

Primers for Region 1

Forward primer TCCCACATTCCATGGGCATC

Reverse primer TGTGCATCTGTTGCATATTGGT

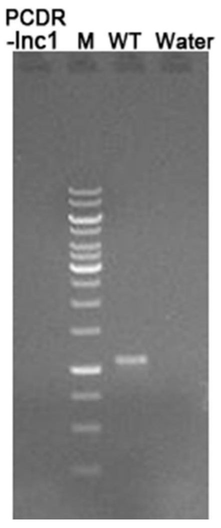

Primers for Region 2

Forward primer TCAACAAACCTCTACCCCCAA

Reverse primer TGGGGCCAACAACTGTAGTG

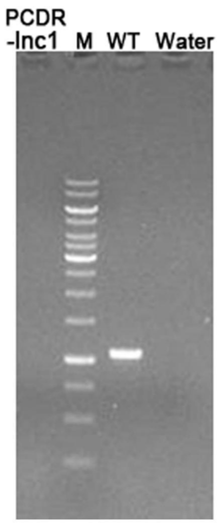

Primers for Region 3

Forward primer CTTCAGAGTCATGTGGCAACAC

Reverse primer TTCTGGCACAATAAGTCTAACAGTC

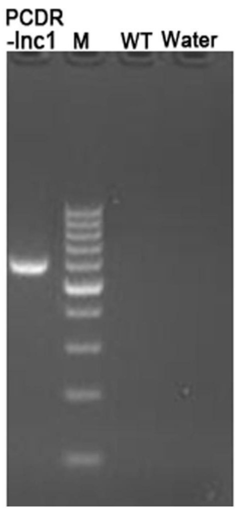

Verification of the deletion of PCDRlnc1 using sequencing

The gene fragments were sequenced and compared before and after knockout. The results are simplified as follows: ATATATATAAAGAGAGAGAGACTTTTTTTTGAAGCCCTGC--del8519 bp----insert 108bp----CCTAGTGGGATGGGAATTGGGGACTGAGAGCGAAGAAGTG

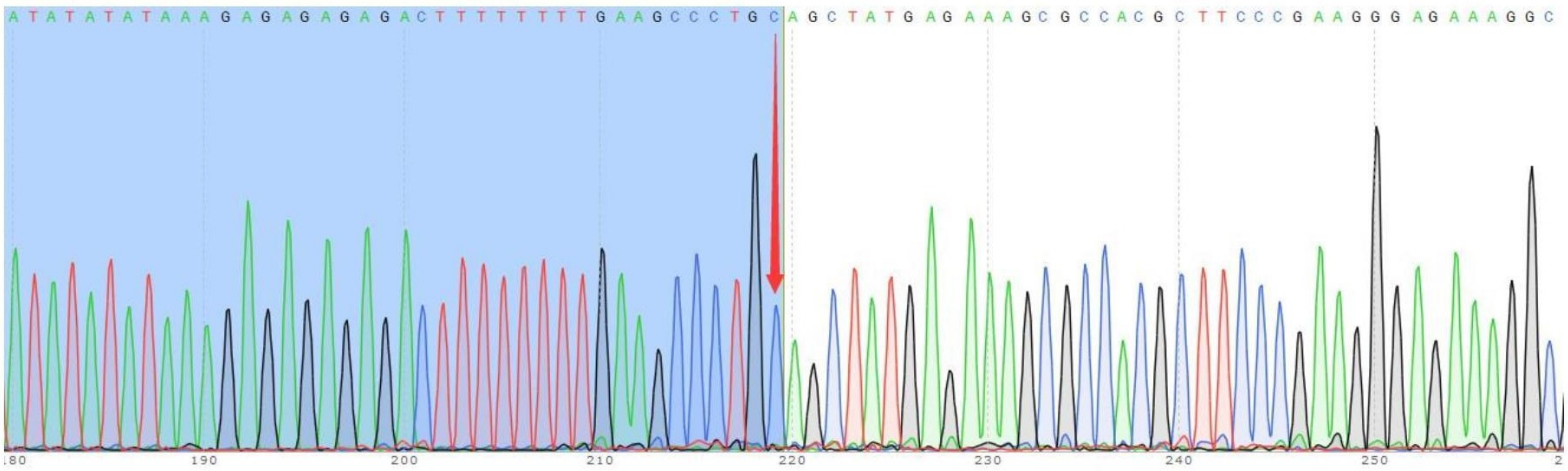

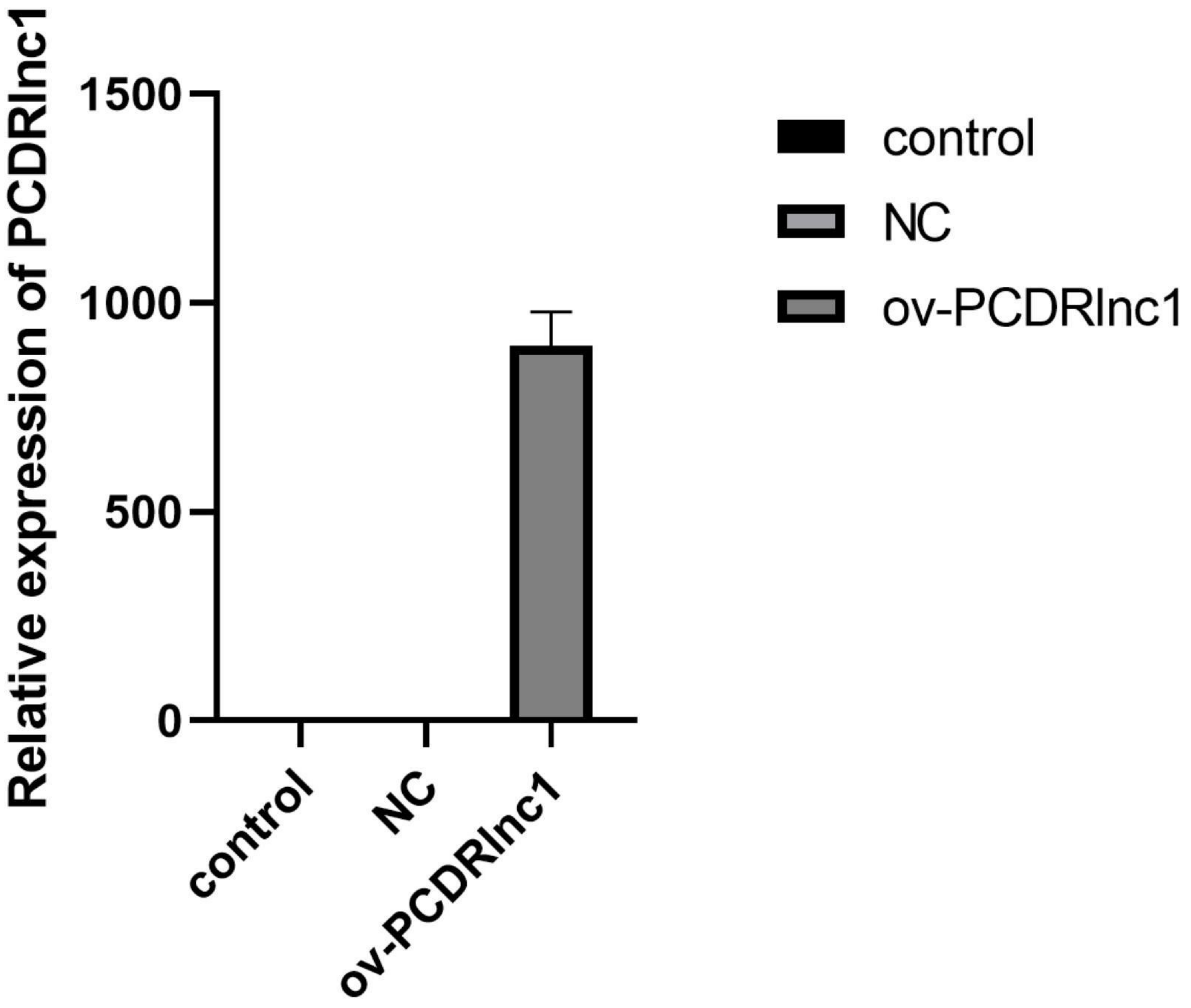
Relative expression of PCDRlnc1
1500
1000
500
0
control
NC
ov-PCDRlnc1
control
NC
ov-PCDRlnc1

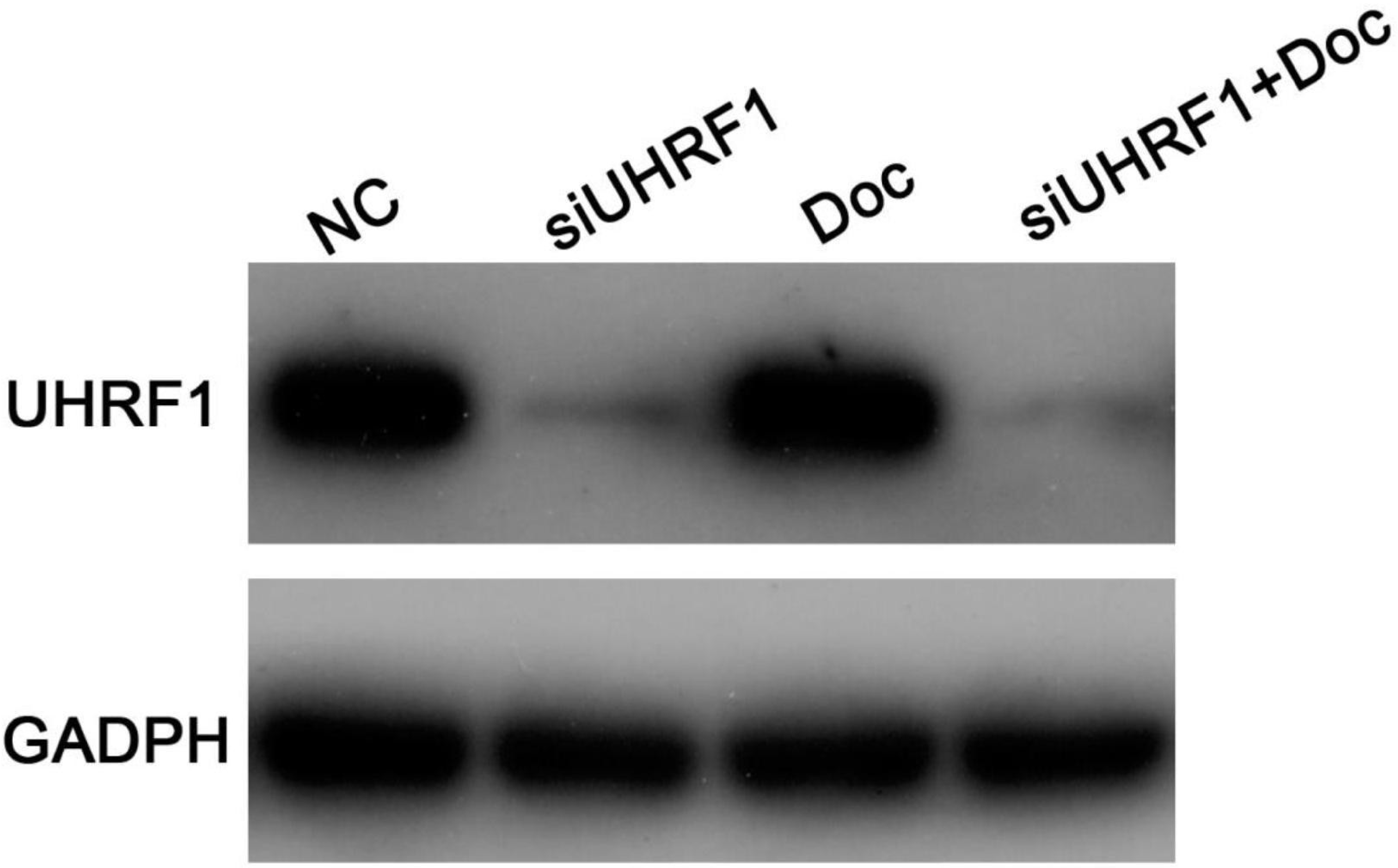
NC
siUHRF1
Doc
siUHRF1+Doc
UHRF1
GADPH
